# Expression of the C-allele of intronic rs8192675 in SLC2A2 is associated with improved glucose response to metformin

**DOI:** 10.1590/1678-4685-GMB-2023-0281

**Published:** 2024-09-20

**Authors:** Wanjun Wang, Suying Chen, Yilei Jiang, Jianhong Ji, Ruochen Cong

**Affiliations:** 1 Shanghai First Maternity and Infant Hospital, Shanghai Key Laboratory of Maternal Fetal Medicine, Shanghai Institute of Maternal-Fetal Medicine and Gynecologic Oncology, School of Medicine, Tongji University, Shanghai, China.; 2 Affiliated Hospital 2 of Nantong University, Department of Radiology, No.666 Shengli Road, Nantong, Jiangsu Province, China.; 3 Affiliated Hospital 2 of Nantong University and First People’s Hospital of Nantong City, Intensive Care Unit, Nantong, People’s Republic of China.

**Keywords:** CRISPR/Cas9, intron, GLUT2, metformin, glucose homeostasis

## Abstract

Glucose is a critical nutrient for energy metabolism. The SLC2A2 gene is essential for glucose sensing and homeostasis, as it encodes the facilitated glucose transporter GLUT2. During diabetes treatment, the C-allele of rs8192675 in SLC2A2 has been found to regulate the action of metformin and reduce the absolute level of HbA1c more effectively than the T-allele. In this study, stable HEK293T cell lines carrying the CC, CT, and TT genotypes of rs8192675 in SLC2A2 were generated using CRISPR/Cas9-mediated genome editing. GLUT2 mRNA and protein levels were elevated in cell clones with the TC genotype compared to those with the CC genotype but were reduced relative to the TT genotype. Additionally, high concentrations of glucose or fructose induced more GLUT2 protein production in CT-genotype cells than that induced in CC-genotype cells, yet less than that induced in TT-genotype cells. Metformin induced a greater increase in GLUT2 expression and a smaller increase in activated AMPK protein expression in CC-genotype cells than those induced in TT-genotype cells, resulting in a remarkable reduction in activated mTOR and S6 levels. This study directly supports the biological mechanism linking the C-allele of rs8192675 with improved treatment outcomes in metformin therapy for diabetes.

## Introduction

Glucose, a major nutrient for energy metabolism, is transported into mammalian cells through sodium-coupled glucose transporters (SGLTs) and glucose transporter facilitators (GLUTs). The human gene for member 2 of the solute transporter family (SLC2A2), also known as GLUT2, encodes 14 GLUT isoforms, each with distinct substrate specificities and cellular localizations (Thorens, 2015). GLUT2, a versatile monosaccharide transporter, transports glucose, mannose, galactose, and fructose, though with low affinity for these substrates (Barahona et al., 2018). As a monosaccharide transporter, GLUT2 is expressed in the plasma membranes of islet beta cells, renal tubule cells, and hepatocytes, among others. It functions as a metabolic glucose sensor and regulates bidirectional glucose transport, balancing the intracellular glucose concentration. GLUT2 is considered a promising target for dietary and pharmacological approaches to regulate intestinal sugar delivery and consequently improve glucose control (Barahona *et al.*, 2018; Wang X et al., 2019).

Mutations in SLC2A2, or GLUT2, can lead to Fanconi-Bickel syndrome, with disorders including glucose and galactose intolerance, hypoglycemia, and hepatorenal glycogen accumulation (Al-Haggar et al., 2011; Grünert et al., 2012). Mammalian SLC2A2 gene knockout showed that GLUT2 mainly modulated glucose reabsorption in the kidney and was required for glucose absorption in the intestine (Schmitt et al., 2017). The rs8192675 variant is located in intron 5 of SLC2A2. In genetic epidemiological research on responses to antihypertensive drugs, this single-nucleotide polymorphism was associated with variations in low-density lipoprotein serum levels (Le et al., 2013). The rs8192675 variant of SLC2A2 affects the efficacy of metformin in the treatment of hyperglycemia in patients with type 2 diabetes (Zhou et al., 2016; Rathmann et al., 2019; Engwa et al., 2020). In patients with diabetes, rs8192675 expression was correlated with a metformin-induced reduction in hemoglobin A1c (HbA1c) levels (Zhou *et al.*, 2016). These studies suggested that the rs8192675 variant of SLC2A2 is functionally intronic.

Metformin is the most commonly used antidiabetic drug, with over 150 million consumers worldwide (He and Wondisford, 2015; Zhou et al., 2018). The drug can reduce long-term blood glucose levels and improve the insulin sensitivity of peripheral tissues without increasing the risk of body weight gain or hypoglycemia. Despite its extensive use over the past 60 years, its precise mechanism of action has not been sufficiently clarified. Metformin affects glucose absorption by regulating sugar transporter expression. It decreases glucose levels by increasing glucose transporter 4 (GLUT4) expression and mediates glucose uptake in skeletal muscles and the absorption of glucose in the intestines (Lv and Guo, 2020). In jejunal brush border membranes, metformin reduced the abundance of SGLT-1 induced by glucose, increased GLUT2 protein level (Sakar et al., 2010), promoted apical GLUT2 localization in rodent enterocytes (Ait-Omar et al., 2011), and concomitantly increased the phosphorylation of intracellular AMP-activated protein kinase (AMPK)α2 (Sakar *et al.*, 2010). Due to the redistribution of glucose transporters in brush border membranes through AMPK control in enterocytes, metformin slightly augmented intestinal glucose absorption. In addition, metformin regulated AMPK expression and promoted mitochondrial fission to enhance mitochondrial respiration and restore the mitochondrial life cycle (Wang Y et al., 2019).

Activated AMPK acts on the signaling of the mammalian target of rapamycin (mTOR) and its downstream effector, ribosomal protein S6 kinase (S6K), which integrates information on nutrient and energy supply and stimulates cell growth and proliferation. The major pharmacological action of metformin is the suppression of mTORC1. Metformin decreased mammalian mTOR activation and improved contractile function in isolated working rat hearts, likely by promoting glucose oxidation. It also acts on tau phosphorylation via mTOR/protein phosphatase 2A (PP2A) signaling in primary neurons (Kickstein et al., 2010). In addition, *in vivo* and *in vitro* studies have shown that metformin exerts its anticancer effect by inhibiting the mTOR/S6K signaling pathway.

In this study, it was demonstrated that metformin increases GLUT2 expression but activates AMPK proteins to a lesser extent in cells carrying the CC genotype than those having the TT genotype of rs8192675. This led to a more pronounced reduction in the activation of mTOR and S6, components of the mTOR signaling pathway. This finding elucidates the variation in metformin’s therapeutic effects across individuals with different rs8192675 variants.

## Material and Methods

### Cell cultivation

Kidney-derived HEK293T cells were procured from the American Type Culture Collection (ATCC, Manassas, VA, USA) and cultured in Dulbecco’s modified Eagle’s medium (DMEM; Gibco, Waltham, MA, USA) containing 10% fetal bovine serum (FBS), 100 µg/mL streptomycin, and 100 µg/mL penicillin in a humidified environment at 37 °C and 5% CO_2_.

### CRISPR/Cas9-mediated editing

For conventional homology-directed repair-mediated gene targeting, CRISPR reagents were generated to target the intron of human SLC2A2 (ENSG00000163581) using the oligos SLC2A2-sgRNA F:5′-CACCGTAGTAGTAGTGGTACTGTAG-3′ and R: 5′-AAACCTACAGTACCACTACTACTAC-3′, and cloned into pSpCas9(BB)-2A-GFP (PX458, which contains the insert hSpCas9; a gift from Dr. Feng Zhang’s lab) to obtain the gene targeting vector PX458-SLC2A2-sgRNA. Donor DNAs harboring sgRNA recognition sites were synthesized with a phosphorothioate modification: 5′-C*A*G*GGAGGGACGAGATGGATGAAGTGGAGGAAGTACAGTAGGGGATGCAATAGTAGTAGTGGTAC**C**GTAGAGGATGAAGTAGATGGGTGCAGTAGGGGATAAGGATGGGGAGTTTTT*A*C*C-3′ and 5′-C*A*G*GGAGGGACGAGATGGATGAAGTGGAGGAAGTACAGTAGGGGATGCAATAGTAGTAGTGGTAC**T**GTAGAGGATGAAGTAGATGGGTGCAGTAGGGGATAAGGATGGGGAGTTTTT*A*C*C-3′(*, phosphorothioate modification; underline indicates the mutation site).

The SLC2A2-sgRNA carrying vectors (15 μg) and donor DNA (5 μg) were dissolved in 800 μL of phosphate-buffered saline (PBS) before being suspended with HEK293T pellets and transfected into HEK293T cells by a modular electroporation system (Gene Pulser Xcell™, Bio-Rad, CA, USA) at 200 V and 500 μF. After 6 h, the cells were resuspended in PBS containing 1% FBS and sorted by flow cytometry to obtain green fluorescent protein (GFP)-positive cells. The sorted GFP-positive HEK293T cells were monocloned in 96-well plates before sequencing the PCR products following amplification using a DNA template from each monoclonal cell and the primer pair rs8192675-F:5 ′-ATCCCACCAACAATTCCAAGG-3′, rs8192675-R:5 ′-CCATCGTCACGGGCATTCTT-3.

### Western blot analysis

The cells were washed with PBS and lysed using a commercial lysis buffer (Solarbio, R0010). After centrifugation at 13,000 × *g* and 4 °C for 20 min, the supernatants were collected, and protein concentrations were measured using a Lowry protein assay. Total cell lysates (20-80 µg protein) were loaded for separation by SDS-PAGE and electro-transferred to a polyvinylidene fluoride membrane. The membrane was incubated with 5% nonfat milk powder in TBST for 2 h at 37 °C before being incubated with primary antibodies at 4 °C overnight.

The antibodies used were anti-GLUT2 (Abcam, cat: ab192599), anti-AMPK-alpha (Cell Signaling Technology, cat: 2532), anti-phospho-AMPK (Thr172) (Cell Signaling Technology, cat: 2531), anti-mTOR (Abcam, cat: ab134903), anti-p-mTOR (Abcam, cat: ab32441), anti-p-S6 (Santa Cruz, cat: sc-20687), anti-S6 (Santa Cruz, cat: sc-271786), and anti-β-actin (Sigma-Aldrich, cat: A5441). Specific peroxidase-conjugated secondary antibodies, or IRDye^®^ 800CW Conjugated Goat (polyclonal) Anti-Mouse (or Rabbit) IgG, were used to detect protein expression using an Odyssey infrared imaging system (LI-COR Bioscience, Lincoln, NE, USA).

### Real-time quantitative reverse transcription PCR (qRT-PCR)

Total RNA was isolated from HEK293T cells using TRIzol reagent (Invitrogen, Carlsbad, CA, USA). During cDNA synthesis, 1 μg of total RNA was reverse-transcribed in a 20 μL reaction using PrimeScript RT Reagent Kits (TaKaRa Bio, Kusatsu, Japan). cDNA samples were amplified using a CFX Real-Time PCR Detection System (Bio-Rad) and SYBR Premix Ex Taq (TaKaRa), according to the manufacturer’s instructions. The primers used were as follows: SLC2A2-F: 5′-CAATGCACCTCAACAGGTAATAAT-3′; SLC2A2-R: 5′- ACCCCATCAAGAGAGCTCCA-3′. The qRT-PCR cycling was conducted with initial denaturation at 95 °C for 50 s, 95 °C for 35 s, and annealing at 60 °C for 36 s for 40 cycles. The 2^−ΔΔCT^ method was used to determine the relative expression, with β-actin as an internal control. Fluorescent signals were measured after each primer-annealing step at 60 °C.

### Immunofluorescence (IF)

Cells were seeded onto glass coverslips, washed with PBS, fixed at 25 °C using 4% paraformaldehyde, and incubated in PBS containing 0.5% Triton-X 100 for 10 min. The cells were then stained with a GLUT2 antibody (Abcam, cat: ab54460), followed by FITC immunoglobulins. Nuclei were counterstained with DAPI. The slides were observed under a confocal laser scanning microscope (CLSM; Leica TCS SP5).

### Statistical analysis

Data are presented as the mean ± standard error of the mean (SEM). All statistical analyses were done using the GraphPad Prism 8 software. Student’s *t* -test was used to evaluate statistically significant differences between two groups, and a One-way Analysis of Variance (ANOVA) was performed to analyze differences among multiple comparisons. A *P* value <0.05 was considered statistically significant.

## Results

### Stable HEK293T cell strains with rs8192675 variants carrying CC, TC, and TT genotypes were obtained using CRISPR/Cas9-mediated editing

**Figure 1 -:**
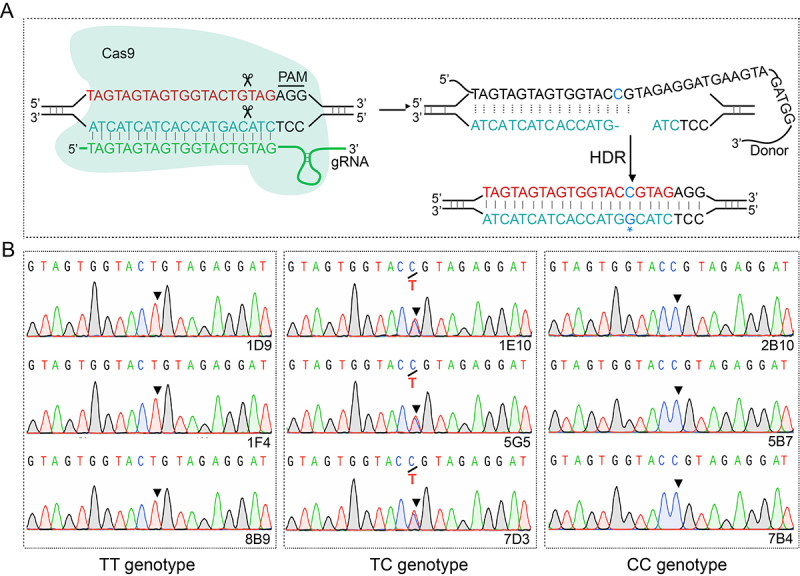
CRISPR-Cas9-mediated gene editing generated cell clones with CC, TT, and TC genotypes at the rs8192675 variant locus. A. Simplified diagram illustrating the principle of gene editing. B. Sanger sequencing of cell clones with CC (2B10, 5B7, 7B4), TT (1D9, 1F4, 8B9), and TC (1E10, 5G5, 7D3) genotypes.

Sanger sequencing revealed that HEK293T cells harbored the TC genotype of the rs8192675 variant. Using CRISPR/Cas9-mediated gene editing, three clones were generated, each carrying the CC genotype (2B10, 5B7, and 7B4; Figure 1B), TT genotype (1D9, 1F4, and 8B9; Figure 1B), and TC genotype (1E10, 5G5, and 7D3; Figure 1B).

**Figure 2 -:**
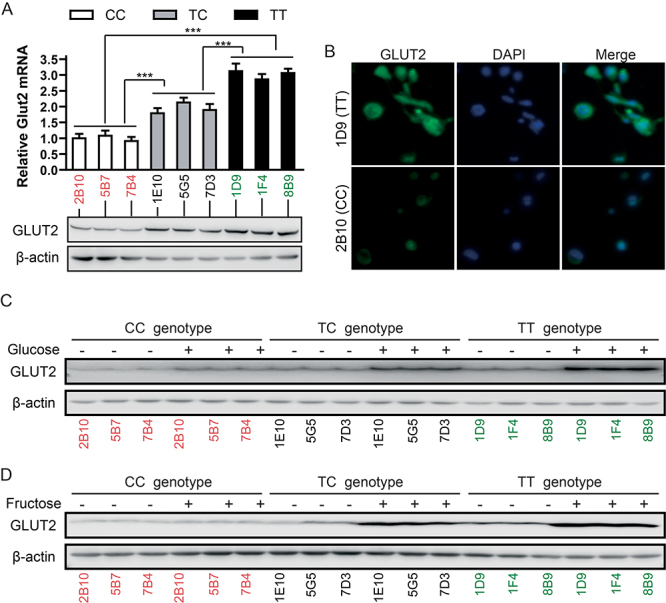
Rs8192675 variant affected GLUT2 protein expression. A. Differences in GLUT2 mRNA and protein expression in cell clones with the CC (2B10, 5B7, 7B4), TT (1D9, 1F4, 8B9), and TC (1E10, 5G5, 7D3) genotypes. B. Immunofluorescence analysis comparing protein expression between 2B9 (CC) and 1D9 (TT), with DAPI labeling of the cell nuclei. C. Effect of high glucose concentration (5 mM) in DMEM on GLUT2 protein expression in the indicated clones (described in A.) after 24 h of incubation. D. Effect of high fructose concentration (5 mM) in DMEM (low glucose) on GLUT2 expression in the indicated clones (described in A.) after 24 h of incubation.

GLUT2 mRNA expression, as determined *via* qRT-PCR, was compared among the CC- (2B10, 5B7, 7B4), TC- (1E10, 5G5, 7D3), and TT-genotype (1D9, 1F4, 8B9) cells, examining the impact of different gene genotypes on the level of mature GLUT2 mRNA in HEK293T cells. It was found that GLUT2 mRNA expression was considerably higher in TC-genotype cells than that in CC-genotype cells, yet remarkably lower than in those with the TT-genotype (Figure 2A, upper panel). Western blot results further confirmed that GLUT2 protein expression was notably higher in TC-genotype cells than that in CC-genotype cells but lower than that in TT-genotype cells (Figure 2A, lower panel). The immunofluorescence analysis also indicated that GLUT2 protein levels were higher in the TT-genotype cell clone 1D9 than those in the TT-genotype 2B10 clone (Figure 2B).

### The rs8192675 variant influences GLUT2 expression, thereby altering the responses to glucose and fructose in HEK293T cells

Given that GLUT2 is a specific transporter that modulates cellular responses to extracellular glucose and fructose levels (Kellett and Brot-Laroche, 2005), the responses of GLUT2 expression to high concentrations of these sugars were analyzed across different genotypes. High concentrations of both glucose and fructose induced higher levels of GLUT2 protein in TT-genotype cells while inducing lower levels in CC-genotype cells than those in TC-genotype cells (Figure 2C, D). These findings indicate that rs8192675 modifies the response of HEK293T cells to elevated extracellular glucose and fructose levels.

### The rs8192675 variant affected GLUT2 expression and changed cellular responses to metformin *via* AMPK-mTOR signaling in HEK293T cells

**Figure 3 -:**
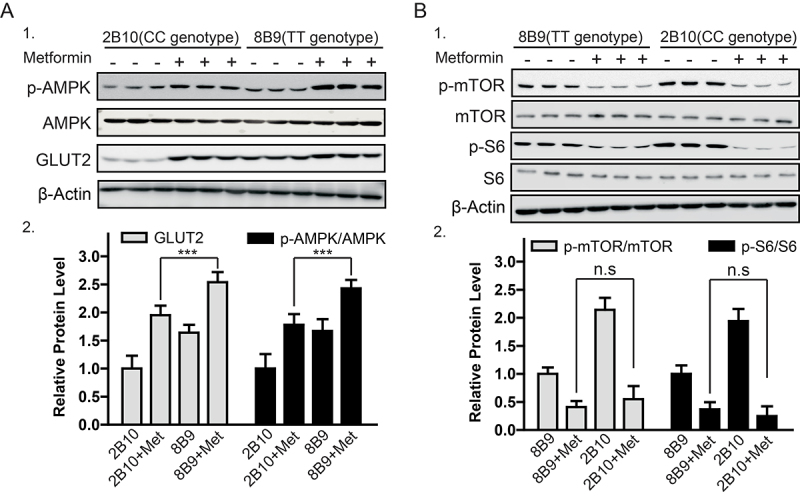
Rs8192675 variant affected GLUT2 expression and changed cellular responses to metformin via AMPK-mTOR signaling in HEK293T cells. A1. Immunoblot analysis of AMPK signaling in clones carrying the CC (2B10) and TT genotype (8B9) in response to metformin (10 µM); A2. Quantified result for A1;***, P < 0.001; B1. Immunoblot analysis of mTOR signaling in clones carrying the CC (2B10) and TT genotype (8B9) in response to metformin (10 µM); B2. Quantified result for B1;***, P < 0.001.

GLUT2 levels affect AMPK activation in humans (Pasula *et al.*, 2023), and metformin affects both AMPK and GLUT2 signaling (Hardie, 2013; Faure et al., 2018). Differences in AMPK activation induced by metformin between the CC and TT cell clones were further compared. CC-genotype cells (2B10) exhibited significantly increased levels of GLUT2 protein and AMPK activation compared to those in the TT-genotype cells (Figure 3A). AMPK has also been reported to regulate mTOR signaling and its associated components (Ali et al., 2017; Chun and Kim, 2021). Therefore, differences in mTOR signaling inhibition induced by metformin between CC- and TT-genotype cell clones were compared. CC-genotype cells induced a greater inhibition of mTOR and S6 phosphorylation by metformin than that in the TT-genotype cells (Figure 3B). These findings could explain the enhanced therapeutic value of metformin in patients with type 2 diabetes possessing the CC genotype (Zhou et al., 2016).

### The rs8192675 variant alters the cellular response to metformin in a dose-dependent manner

**Figure 4 -:**
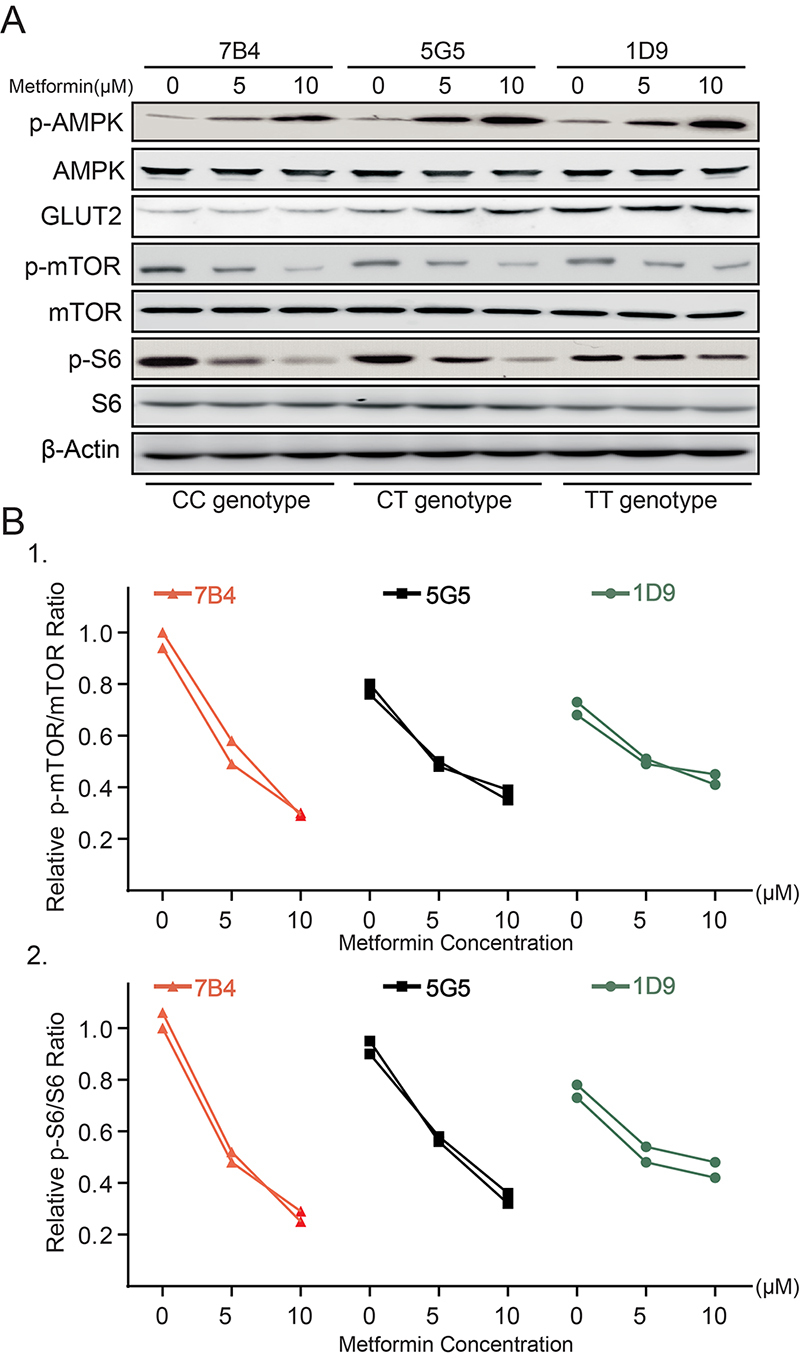
Rs8192675 variant changed cellular response to metformin in dose-dependent manner. A. Immunoblot analysis of AMPK and mTOR signaling in clones carrying the CC (7B4), TC (5G5), and TT genotype (1D9) in response to the indicated dose of metformin; B1 and B2. p-mTOR/mTOR and p-S6/S6 ratios from two representative experiments.

It was observed that the regulation of AMPK and mTOR signaling by metformin depends on the rs8192675 polymorphism and exhibits dose-dependency. Western blot analysis confirmed that 10 µM metformin induced greater activation of AMPK in 7B4 clones (CC genotype) than that in 5G5 (TC genotype) and 1D9 (TT genotype) clones (Figure 4A). As the concentration increased, metformin exhibited a stronger inhibition of mTOR and S6 signaling in the CC-genotype than that in the TC- and TT-genotype cells (Figure 4A, B).

## Discussion

Metformin, commonly prescribed for the treatment of type 2 diabetes, exhibits considerable variability in efficacy and occasionally serious adverse reactions (Bailey and Turner, 1996; Florez, 2017). Previous studies have demonstrated that patients treated with the drug who carry the C-allele in rs8192675 of SLC2A2 experience a remarkable reduction in blood glucose levels (Zhou et al., 2016; Rathmann et al., 2019). This study investigates the mechanisms by which the rs8192675 variation influences the therapeutic efficacy of metformin.

The SLC2A2 gene encodes GLUT2, a glucose transporter highly expressed in the human liver, small intestine, kidneys, and islets (Thorens, 2015). The rs8192675 of the SLC2A2 gene correlates with a higher prevalence of diabetes symptoms, though this variation was not localized in SLC2A2 mice. To investigate the potential mechanism of rs8192675 variation in altering the therapeutic efficacy of metformin, HEK293T cell clones carrying the CC, TT, and TC genotypes at rs8192675 were established using CRISPR/Cas9 gene editing (Figure 1). These cells, derived from kidney-originated HEK293 cells, are rich in GLUT2 proteins. Although several studies have suggested that the C-allele of rs8192675 affects the expression of SLC2A2 (Soranzo et al., 2010; Scott et al., 2012), the findings from this study confirm that the rs8192675 variant carrying the CC genotype had the lowest expression of GLUT2, whereas the TT genotype had the highest GLUT2 expression (Figure 2A, B). For several decades, introns have been considered essential in the regulation of gene expression (Kumari et al., 2022). The results from this study exemplify how intron variations can influence gene expression, highlighting the need for further research to fully elucidate the mechanism.

The rs8192675 polymorphism is implicated in genetic defects in glucose metabolism in type 2 diabetes (Zhou et al., 2016). The proportion of patients carrying the CC genotype is considerably lower than those with the TT genotype, possibly due to long-term evolutionary selection in humans. In individuals with obesity, altered GLUT2 localization in enterocytes has been observed compared to non-obese counterparts (Ait-Omar et al., 2011). Furthermore, it was observed that the CC genotype in rs8192675 results in decreased cellular GLUT2 protein levels (Figure 2), which may also reduce GLUT2 localization to the cell surface, thereby altering glucose uptake.

As the cellular glucose and energy sensor, AMPK has a major role in the mechanism of metformin action (Steinberg and Hardie, 2023). A previous study showed that AMPK activation induced an increase in glucose uptake by GLUT2 (Walker et al., 2005). Metformin was observed to increase GLUT2 expression in cells with the CC genotype at the rs8192675 locus (Figure 3A), consistent with previous findings in other tissues (Ait-Omar et al., 2011; Wang et al., 2018). The fact that the CC genotype further promoted the activation of AMPK and inhibition of mTOR and S6 phosphorylation by metformin compared with that in the TT variant (Figure 3B, Figure 4) suggests that decreased GLUT2 protein levels may promote AMPK activation and that metformin provides dual regulation between the levels of GLUT2 protein and AMPK activity.

To obtain more solid conclusions regarding the impact of the rs8192675 variation on metformin treatment efficacy, further studies are planned using cells from human pancreatic islets and livers or humanized SLC2A2 transgenic mice. The findings indicated that the CC genotype in rs8192675 affects GLUT2 expression, thereby influencing the regulation of the cellular AMPK and mTOR signaling pathways in response to metformin. This variability could account for the differing therapeutic effects of metformin among individuals with distinct rs8192675 variants. The results also suggest that metformin may maintain glucose homeostasis by restoring GLUT2 expression.
